# Measuring Biochemical Variables and Serum Amyloid A (SAA) in Working Mules in Central Italy

**DOI:** 10.3390/ani12202793

**Published:** 2022-10-16

**Authors:** Marilena Bazzano, Francesca Arfuso, Laura Bonfili, Anna Maria Eleuteri, Amy McLean, Evelina Serri, Andrea Spaterna, Fulvio Laus

**Affiliations:** 1 School of Biosciences and Veterinary Medicine, University of Camerino, 62032 Matelica, Italy; 2 Department of Veterinary Sciences, University of Messina, 98122 Messina, Italy; 3 Department of Animal Science, University of California Davis, Davis, CA 95616, USA

**Keywords:** fieldwork, mule, acute phase response, serum amyloid A, biochemical profile

## Abstract

**Simple Summary:**

Mules are thought to be stoic animals that generally express few signs of illness or disease. Mules used as working equids in strenuous conditions on a daily basis may not show clinical signs of disease until the disease is more advanced. The help of stable side tests and measuring variables such as acute phase proteins, which are known to elevate in a rapid manner due to infection or inflammation, may assist owners and practitioners with detecting early signs of disease in working mules. This study measured serum amyloid A (SAA), an acute phase protein, in 10 healthy working mules in Central Italy. The study found no change in SAA for mules working 8 h but mild increases in serum electrolytes, urea, and creatinine concentrations that supported a loss of body water and decreased renal blood flow in response to exercise.

**Abstract:**

According to FAO reports, the global mule population counts about 9 million mules. This hybrid cross of a male donkey and a female horse is mainly used for draft purposes because they are thought to be strong and hardy animals. Most consider mules to be less susceptible to disease and fatigue compared to horses. Therefore, the aim of this study was to investigate the effects of fieldwork on biochemical variables and serum amyloid A in working mules. Blood samples were collected from 10 healthy, female, working mules before and after 8 h of fieldwork. According to statistical analysis, a significant influence (*p* < 0.05) of fieldwork was found on mules’ electrolyte profile with increased levels of sodium, chloride, and calcium, as well as on blood urea nitrogen and creatinine. After a day of fieldwork, serum sodium, chloride, calcium, urea, and creatinine concentrations were increased, supporting decreases in body water and renal blood flow. However, without comparison to a group of mules that were not exercised yet maintained under similar ambient conditions, it is uncertain whether these changes can be attributed to exercise. Further, no change in SAA concentration was found after exercise, indicating that the work performed did not result in systemic inflammation.

## 1. Introduction

The mule (*Equus mulus*) is a hybrid cross of a male donkey (*Equus asinus*) and a female horse (*Equus caballus*). The world population of mules is estimated to be close to 9 million animals, which are found primarily as a beast of burden thanks to their strength and rusticity, but more mules are being used as performance and recreational animals [1]. These working equids are the foundation of many developing countries by providing strength, utility, and endurance to support labor and agriculture [2].

In both low- and middle-income countries, working equids continue to be an essential component of the livelihoods of millions of families worldwide. These animals are often considered at risk for their welfare since the countries where they live are characterized by poor resources in terms of food and water availability [3,4]. Furthermore, the context of the countries should also be considered: for example, the lack of roads, the isolation of communities, and the cultural and religious situation that may influence working equid welfare and assessment [5,6].

The arduous activities working equids are subjected to can often compromise the overall animal health and welfare, making animals more susceptible to disease, thus increasing the tendency for sickness and decreasing their ability to work efficiently [2].

Poor body condition score (BCS), high incidence of musculoskeletal disorders, skin lesions associated with the harnessing system, dental diseases, parasitosis, and behavioral disorders, such as apathetic behavior or aggressivity, are often detected in working equids such as mules [4].

Heavy work, such as physical activity, is a commonly recognized stress factor that can perturb the homeostasis of an organism [7,8]. Blood profiles assess an animal’s internal environment highlighting possible impairment in homeostasis as evidenced by marked fluctuations in hematological indices following several physiological or pathological conditions [9,10].

These variables can be used as tools for assessing the appropriateness of the workload they are requested to perform. The study of biochemical changes resulting from physical activity allows researchers to deeply understand the adaptations occurring within the animal’s body following physical effort and to adopt suitable measures to preserve animal welfare and health status [10,11,12].

It is widely accepted that physical activity elevates circulating acute phase protein (APPs) levels also in equine species [12,13]. Tissues under heavy physical activity, similarly to infection conditions or inflammation, initiate a stereotyped sequence of defense reactions known as acute phase response (APR) [14]. The onset of an APR is correlated with the duration and intensity of physical exercise. The dynamic process of APR involves systemic metabolic changes, part of the systemic non-specific defense, before triggering a specific immune response [15]. The complicated but precise regulation network of APR is orchestrated by the pro-inflammatory mediators, among which the cytokines play very important roles [15,16]. The cytokines initiate the APR cascade through the stimulation of several cell types. A central pathophysiological step of the APR is hepatic synthesis modulating the circulating concentrations of some APPs, known as positive and negative APPs, according to their fluctuation after the onset of APR. Among acute phase proteins, serum amyloid A (SAA) is considered one of the most valuable biomarkers of the APR in equids as it tends to increase up to 100-fold following pathological conditions such as inflammation, infection, neoplasia, trauma, and stressful events such as physical exercise [2]. However, little is known about APR and APPs in mules, which have been explored in sick working animals and after vaccination [2,17].

Due to the stoic nature and limited information on blood chemistry in mules, conditions such as strenuous field work or exercise can result in damage to the body, which can go undetected until advanced stages develop and the animal is no longer able to perform its daily tasks.

Mules are thought to be less susceptible to diseases and fatigue compared to horses, yet no scientific investigation has been performed so far to support this belief. In view of such considerations, the purpose of the current survey was to investigate the effects of strenuous exercise on health status and welfare by evaluating SAA and biochemical variables in working mules in Central Italy.

## 2. Materials and Methods

### 2.1. Animals and Experimental Groups

Ten adult female mules, with a mean age (±standard deviation) of 15 ± 5 years and a mean weight (±standard deviation) of 450 ± 40 kg, were included in the study. The body condition score (BCS) of animals ranged from 2 to 3 out of 5 [18]. Owner’s consent was given for all animals included in the study.

The mules were reared in Central Italy as working animals and were kept in outdoor paddocks. The management was the same for all the mules, their diet consisted of fresh forage (grass), 8 ± 1 kg grass hay and 4 ± 0.5 kg concentrates per day, and water ad libitum.

**Figure 1:**
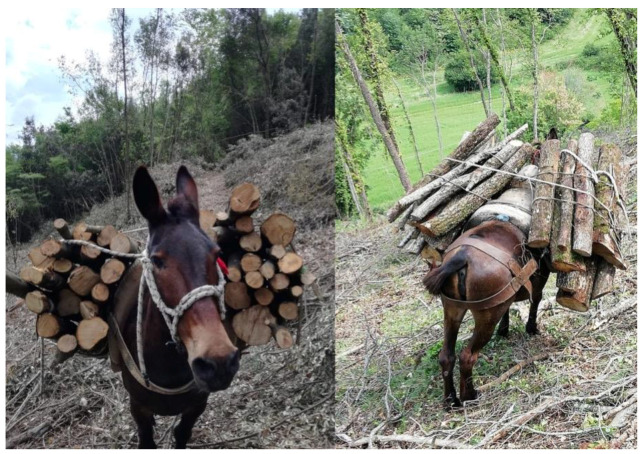
Working mules carrying wood. The use of an appropriate harness can easily avoid the occurrence of skin lesions due to wood weight.

The animals were routinely used for wood transportation in a hill country 5 days per week during the spring season. Each animal carried 200 ± 10 kg of wood. The mules walked 60 km on a path with a mean slope of 15 ± 5%. Animals walked about 30 km unloaded to gather wood and then walked backloaded (Figure 1).

Blood samples were collected, by jugular venipuncture, on the first working day of the week in the morning (08:00 AM, T0) at rest before taking the mules out of paddocks and in the afternoon (04:00 PM, T1) immediately after the work was completed. Weather conditions during the work and blood sampling were as follows: average temperature 13 °C (11–14 °C), average relative humidity 86% (76–93%), and partially cloud weather. During the work period, mules had the opportunity of eating some grass while loading the wood and had access to water four times during the daily working period. All mules were regularly vaccinated, checked for intestinal parasitosis, and treated when necessary.

Animals were clinically evaluated before and after work with particular attention to musculoskeletal injuries, skin lesions, and behavioral alterations.

All animal housing, care, and experimental procedures herein described were approved by the Animal Care Committee of Camerino University (Registration number: E81AC.8.B, 1 March 2018) and were in accordance with the standards recommended by the EU Directive 2010/63/EU for experiments on animals.

### 2.2. Biochemical Profile and Serum Amyloid Analysis

After collection, blood samples were placed on ice and delivered to the laboratory within 2 h. At the lab, samples were centrifuged for 10 min at 1000× *g* (Universal 32, Hettich Zentrifugen, Germany), and the obtained sera were divided into two 1.5 mL aliquots and stored at −20 °C until analysis. Sera were tested for potassium (K), sodium (Na), chloride (Cl), calcium (Ca), phosphorus (P), calcium/phosphorus ratio (Ca:P) blood urea nitrogen (Bun), γ-glutamyl transferase (GGT), glucose (Glu), creatinine (Cre), glutamic oxaloacetic transaminase (Got), serum glutamic pyruvic transaminase (GPT, lactate dehydrogenase (LDH), alkaline phosphatase (ALP), cholesterol (Chol), triglyceride (TG), creatine kinase (CK), total bilirubin (TBil), direct bilirubin (DBil), indirect bilirubin (IBil), total protein (Tp), albumin (Alb), globulins (Glob), and albumin/globulin ratio (A/G) by using the automatic clinical chemistry analyzer BT 3500 VET plus (Biotecnica Instruments, Rome, Italy).

Serum amyloid A (SAA) was detected in serum samples by a solid phase sandwich enzyme-linked immunosorbent assay (ELISA) using the Tridelta PhaseTM range SAA kit (Tridelta Development Ltd., Maynooth, Ireland). Briefly, a monoclonal antibody specific for SAA was coated onto the wells of the microtiter strips provided. The 1:2000 diluted samples and calibrators of known SAA content were incubated in micro-wells at 37 °C together with horseradish peroxidase (HRP)-labeled anti-SAA antibody. Any SAA present was captured between the coated microplate and the labeled antibody. After the washing steps, the chromogenic substrate 3,3′,5,5′-tetramethylbenzidine was added. The resulting blue product is directly proportional to the amount of SAA present in the serum of mules. The reaction was stopped by adding a stop solution, and the intensity of color was measured at 450 nm using a microtiter plate reader. The concentrations of the test samples were derived from the calibration semi-logarithmic standard curve and were expressed as mean concentration (µg/mL) ± SE. Intra (within)-assay precision/reproducibility was 4.4–5%, whereas inter-batch assay reproducibility was 6.22–11.4%.

### 2.3. Statistical Analysis

Data were analyzed using the statistical software Prism 8 (Graphpad Software Ltd., San Diego, CA, USA). A Shapiro–Wilk test was used to verify the normal distribution of data. Paired Student’s *t*-tests and Mann–Whitney test were used for normally and not normally distributed data, respectively. *p*-values were reported to show significant differences in studied blood variables before (T0) and after (T1) the working section. Values of *p* < 0.05 were considered statistically significant.

## 3. Results

All the mules were accustomed to the type of exercise and completed the working section in about 8 h. None of the animals showed any sign of disease, lameness, skin lesions, or behavioral alteration during the experimental period. Mean values and standard deviations of biochemical variables are shown in Table 1.

According to statistical analysis a significant influence of fieldwork was found on mules’ electrolyte profile with mean levels of sodium (*p* = 0.048) ranging from 137 mEq/L (T0) to 140 mEq/L (T1), chloride (*p* = 0.014) ranging from 100 mEq/L (T0) to 104 mEq/L (T1), calcium (*p* = 0.001) ranging from 12.5 mg/dL (T0) to 13.7 mg/dL (T1), while mean phosphorus decreased (*p* = 0.02) from 4 mg/dL to 3.6 mg/dL, with consequent modification in Ca/P ratio (*p* = 0.001) that passed from 3.1 to 3.8. Significant increases in the concentrations of some biochemical variables such as Bun (*p* = 0.03), Cre (*p* = 0.0003) and Glu (*p* = 0.0001) have been observed as well. On the contrary, SAA did not statistically change (*p* = 0.85) in mules during burden work, ranging from 4.84 μg/mL at T0 to 5.41 μg/mL at T1.

## 4. Discussion

Heavy physical activity could represent a stressful condition capable of disturbing an animal’s homeostasis, inducing alterations in several body systems. Systemic changes occurring during work are reversible thanks to the activation of key regulatory systems that cooperate to reestablish homeostatic equilibrium [7,8,19,20]. The failure of the organism to restore balance can have a negative impact on animal health status [19]. According to the results obtained in the current study, fieldwork seems to influence the mules’ macromineral and electrolyte profile by determining increased levels of sodium, chloride, and calcium, suggesting a fluid shift from serum that establishes a change in electrolytic balance likely to occur during strenuous physical activity [21,22]. However, these minor changes (2–3% increase in sodium and chloride concentrations) could simply have been due to diurnal, ambient conditions, or feeding effects and may not totally be attributable to the work performed. A mild body water deficit (2–3%) sustained over the course of the 8 h of work could also contribute to this effect on sodium and chloride serum concentrations.

Interestingly, the mules included in this study showed increased levels of calcium and decreased levels of phosphorus after field burden work, as well. This finding differs from previous surveys carried out on exercising horses that found a decrease in blood calcium after physical effort [22] due to a decreased production of parathyroid hormone by parathyroid glands [23]. This difference might be due to the different types of exercise; in fact, according to a study by Vervuert and colleagues [24], draft load exercise with low velocities had no effect on Ca metabolism. Therefore, the increase in calcium herein observed might be the result of hemoconcentration and the type of physical effort such as the fieldwork. At the same time, the work performed by the mules caused a reduction in P and then an increase in C/P, as observed after work.

A significant increase in the concentrations of Bun and Cre was observed in mules after the draft work. These findings agree with previous observations on the trend of these variables in horses after physical effort [25]. Bun and Cre are traditional indices of renal function and could also be affected by prerenal factors, such as hemoconcentration. The increase in Bun could be associated with the higher rate of protein breakdown caused by muscular activity on draft performance. Additionally, creatinine increases during exercise as a result of increased phosphocreatine turnover, so this increase cannot be used as an indicator of a reduced glomerular filtration rate.

While it has not been described in other equids, increased serum levels of gamma glutamyl transferase (GGT) are linked to overtraining syndrome or maladaptation to exercise in horses [26,27]. In this study, GGT levels decreased in mules after the work session, which may reflect a positive adaptation to work. Further studies elucidating the response to working and overworking in mules are needed.

Glucose tends to decrease during and after physical activity in horses [28], yet this study showed the opposite, and glucose increased in mules after work. The glucose response difference observed in mules in this study may be related to a difference in physical activity or a difference in equid physiology, and future studies are needed for further investigation into glucose response in mules post work.

Creatine kinase did not increase in this group of mules, demonstrating that the workload (distance, time of work, weight of the wood) was well tolerated and not excessive.

The use of APPs in veterinary clinical chemistry as non-specific variables for monitoring inflammatory activity has been adopted during high-intensity physical activity, as the occurrence of muscle injury can trigger the APR [29].

The physiological response to exercise differs in acute response and chronic adaptation. It has been shown that there is an analogous relationship between the APR to exercise and existing infectious and inflammatory processes so that the presence of any tissue injury promotes the release of pro-inflammatory cytokines, nitric oxide, and glucocorticoids that activate and modulate the APR and release of APPs, primarily by the liver [30]. The determination of these proteins could be, therefore, useful in monitoring the health status of the animal [31]. It is widely accepted that physical and psychological stress elevates circulating APPs levels both in experimental animals and horses [13,32,33]. Although differences exist for separate proteins among species, the positive APPs of domestic animals can be listed in three major groups: positive APPs showing an increase of about 50%, including ceruloplasmin and complement factor-3; positive APPs showing an increase of 2–3-fold including haptoglobin, fibrinogen, α-globulins with antiprotease-activity and lipopolysaccharide-binding protein, and, finally, positive APPs displaying a rapid increase of up to 5-fold to 1000-fold including C-reacting protein (CRP) and SAA. Although the function of most APPs has not been totally elucidated, it is well established that the positive APPs are regarded as having general functions in opsonization and trapping of micro-organisms and their products, in activating complement, in binding cellular remnants such as nuclear fractions, in neutralizing enzymes, scavenging radicals, and in modulating the host’s immune response. Therefore, the acute phase reaction offers a biological effect mechanism appropriate to include in future systems for assessing health status both in animals and human patients. Among positive APPs, the SAA, an apolipoprotein of high-density lipoprotein, is thought to influence high-density lipoprotein-cholesterol transport. In tissues, it attracts inflammatory cells, inhibits the respiratory burst of leukocytes, and modulates the immune response [2,29,31].

There is a paucity of studies on SAA concentrations in mules, and preliminary data indicated a difference in SAA response in mules versus horses or donkeys (Table 2), suggesting that potential variables specific to a hybrid species may impact the inflammatory response rate or SAA concentration over time [2,32,33,34,35].

The circulating values of SAA obtained in the present investigation ranged from 4.84 μg/mL at T0 to 5.41 μg/mL at T1 in the investigated mules, suggesting that draft work did not significantly affect the APR in mules that are accustomed to and regularly trained for this type of fieldwork. These findings seem to indicate that mules can cope with strenuous exercise, which did not promote enough physiological stress to cause an increase in the concentration of SAA in mules, thus suggesting a great ability of mules to adapt to fieldwork. However, the absence of a control group might represent a limitation of the study as the differences herein reported may be related to or moderated by the temporal effect.

## 5. Conclusions

Mules have supported man for millennia and are currently critical to the lives of rural developing countries and have taken on a new role as valued performance and recreational animals in industrial countries. There is potential for working mules to suffer from a poor harness, lack of veterinary care, improper nutrition, and low status and value, despite their usefulness. Because of this important role as working equids, the scientific community should direct attention to improving the knowledge about the assessment of mules’ welfare. Such studies can also be helpful for clinicians treating mules in developing countries and further develop their understanding of biochemical and inflammatory responses in mules.

It is well known that the welfare status of working equids is generally poorer than other equids with different attitudes [36]. Our findings suggest that fieldwork performed in this study by mules does not interfere with the homeostatic equilibrium of animals’ body systems, as demonstrated by the lack of inflammatory response measured by SAA levels. Suitable management of animals in terms of sustainable workload, feeding, human-animal relationship, routinary control of health status, and application of preventative medicine confirm an ethical use of animals where mechanical vehicles are not suitable.

Although the authors are aware of the difficulties that can be encountered in developing countries in observing certain good practices, this work demonstrates how the correct management of animals is compatible with their use as working animals.

Further studies are advocated to better evaluate other possible markers of inflammation and/or stress in these working equids to control and preserve their welfare and health status.

## Figures and Tables

**Table 1:** Mean values ± standard deviations (SD) of studied variables in mules before and after work. *p*-values obtained by paired *t*-test and Mann–Whitney test (*), respectively, are indicated in bold when statistically significant.

Variable	Unit	Before Work	After Work	*p*-Values
SAA *	ug/mL	5.4 ± 5.2	4.8 ± 3.2	0.85
K	mEq/L	4.0 ± 0.3	3.8 ± 0.3	0.18
Na	mEq/L	136.9 ± 4.7	139.7 ± 1.9	**0.048**
Cl	mEq/L	100.4 ± 3.5	103.4 ± 1.9	**0.014**
Ca	mg/dL	12.6 ± 0.7	13.7 ± 0.7	**0.001**
Bun	mg/dL	33.1 ± 4.9	37.0 ± 3.5	**0.03**
GGT	UI/L	29.7 ± 4.8	23.5 ± 4.3	**0.003**
Alp	IU/L	546.5 ± 106.4	528.6 ± 103.2	0.35
Cre	mg/dL	1.2 ± 0.1	1.4 ± 0.1	**0.0003**
Glu	mg/dL	76.3 ± 9.0	95.5 ± 6.3	**0.0001**
LDH	UI/L	229.3 ± 68.6	227.7 ± 68.8	0.47
Chol *	mg/dL	100.3 ± 10.6	102.0 ± 11.0	0.75
TG	mg/dL	27.4 ± 8.8	28.0 ± 3.4	0.41
Ck *	UI/L	232.8 ± 65.9	247.6 ± 212.7	0.22
Got *	UI/L	104.7 ± 33.4	98.8 ± 41.1	0.56
GPT	UI/L	2.1 ± 1.1	2.6 ± 1.4	0.18
Alb	g/dL	3.2 ± 0.2	3.3 ± 0.2	0.32
P	mg/dL	4.1 ± 0.5	3.6 ± 0.4	**0.02**
TP	g/dL	8.3 ± 0.3	8.3 ± 0.4	0.47
DBil	mg/dL	0.3 ± 0.1	0.3 ± 0.0	0.32
TBil	mg/dL	0.8 ± 0.1	0.7 ± 0.1	0.19
IBil	mg/dL	0.5 ± 0.1	0.4 ± 0.1	0.14
C/P	ratio	3.1 ± 0.4	3.8 ± 0.6	**0.001**
Glob	g/dL	5.0 ± 0.4	5.0 ± 0.5	0.48

**Table 2:** SAA values reported in horses (H), donkeys (D), and mules (M) with different conditions.

Species	SAA (μg/mL)	Condition	Reference
Mixed (H,D,M)	688–4000	Systemic inflammation	[2]
Mixed (H,D,M)	1.8–14.5	No inflammation	[2]
Donkey (adults)	2.38 ± 1.78	Lactation (healthy)	[34]
Donkey (adults)	25.95 ± 14.98	Parturition (healthy)	[34]
Donkey (foals)	37.44 ± 19.75	Healthy newborns (<48 h)	[34]
Equine (adults)	0–20	Healthy	[35]
Equine (foals)	<30	Healthy newborns	[35]

## Data Availability

Not applicable.
